# Molecular, biochemical, and clinical analyses of five patients with carbamoyl phosphate synthetase 1 deficiency

**DOI:** 10.1002/jcla.23124

**Published:** 2019-11-20

**Authors:** Lijuan Fan, Jing Zhao, Li Jiang, Lingling Xie, Jiannan Ma, Xiujuan Li, Min Cheng

**Affiliations:** ^1^ Department of Neurology Children's Hospital of Chongqing Medical University Chongqing China; ^2^ Ministry of Education Key Laboratory of Child Development and Disorders Chongqing China; ^3^ China International Science and Technology Cooperation Base of Child Development and Critical Disorders Chongqing China; ^4^ Chongqing Key Laboratory of Pediatrics Chongqing China

**Keywords:** carbamoyl phosphate synthetase 1 deficiency, clinical presentation, CPS1, hyperammonemia, urea cycle disorders

## Abstract

**Background:**

Carbamoyl phosphate synthetase 1 deficiency (CPS1D) is a rare urea cycle disorder. The aim of this study was to present the clinical findings, management, biochemical data, molecular genetic analysis, and short‐term prognosis of five children with CPS1D.

**Methods:**

The information of five CPS1D patients was retrospectively studied. We used targeted next‐generation sequencing to identify *carbamoyl phosphate synthetase 1* (*CPS1*) variants in patients suspected to have CPS1D. Candidate mutations were validated by Sanger sequencing. In silico and structure analyses were processed for the pathogenicity predictions of the identified mutations.

**Results:**

The patients had typically clinical manifestations and biochemical data of CPS1D. Genetic analysis revealed nine mutations in the *CPS1* gene, including recurrence of c.1145C > T, five of which were firstly reported. Seven mutations were missense changes, while the remaining two were predicted to create premature stop codons. In silico and structure analyses showed that these genetic lesions were predicted to affect the function or stability of the enzyme.

**Conclusion:**

We reported five cases of CPS1D. Five novel mutations of *CPS1* gene were found. Mutations of *CPS1* have private nature, and most of them are missense compound heterozygous. The mutation affecting residue predicted to interfere the catalytic sites, the internal tunnel, or the regulatory domain results in severe phenotype.

## 1 INTRODUCTION

The urea cycle (UC) occurring in the liver is the only pathway capable of metabolizing waste nitrogen. It mainly consists of five catalytic enzymes, including carbamoyl phosphate synthetase 1 (CPS1), ornithine transcarbamylase (OTC), argininosuccinate synthetase (ASS), argininosuccinate lyase, and arginase, and a cofactor producer N‐acetylglutamate synthase (NAGS).^1^ Carbamoyl phosphate synthetase 1 (CPS1) deficiency (CPS1D; MIM# 237300; EC 6.3.4.16) is a genetic disorder of the CPS1 enzyme, which is inherited as an autosomal recessive trait.^2^ The CPS1 enzyme catalyzes the first step of the UC, and during this process, ammonia is converted into carbamoyl phosphate.^3^ The incidence of CPS1D has been reported to be 1 in 1 300 000.^4^ Mutations of the *CPS1* gene may cause partial or total absence of the enzyme activity, which can hamper the detoxification of excess nitrogen resulting from breakdown of protein, lead to acute hyperammonemia (HA), and present as a series of neurological malfunctions.^5^

Traditionally, CPS1D has been classified into two phenotypes. Individuals with CPS1D range from undetectable to a residual activity of ≤5% may have a neonatal onset,^6^ presenting as somnolence, poor feeding, vomiting, hypothermia, hyperventilation, and seizures that rapidly progress to lethargy and coma that correspond with accumulation of ammonia and other precursor metabolites during the newborn period.^7^, ^8^ Delayed recognition and measurement to drop blood ammonemia could cause permanent damage to the central nervous system or even death. Furthermore, late‐onset CPS1D can occur at any period of life after some forms of environmental stress, for example, infection, increased dietary protein intake, applying certain drugs, pregnancy, surgery, or without discernible cause.^6^, ^9^ These events could arouse a hypermetabolic state or interfere with the enzyme's function resulting in metabolic decompensation. The phenotype of late‐onset CPS1D is less severe and more heterogeneous, including headache, behavioral or psychiatric problems, learning disabilities, periodic vomiting, and vegetarian, and in acute and severe cases, it could manifest as stroke, seizures, and death.^1^, ^9^

Since clinical manifestations are nonspecific, CPS1D is prone to be misdiagnosed in the hospital. The diagnosis of CPS1D is primarily based on the clinical presentation, biochemical, and genetic testing. In healthy individuals, blood ammonia concentration is maintained at 10‐40 μmol/L. Ammonia >60 μmol/L can cause digestive and neurology symptoms, such as anorexia, nausea, excitement, and insomnia.^10^ Low levels of blood citrulline, high levels of blood glutamine, and no increase in urinary orotic acid are characteristic biochemical changes in CPS1D.^11^, ^12^ The enzyme's proper function requires N‐acetylglutamate (NAG) as an allosteric activator, dysfunction of which can result in the same clinical and laboratory findings. Therefore, genetic analysis is a key element in diagnosing CPS1D and for performing counseling.^12^

The human *CPS1* gene is located on chromosome 2q35. It is a large gene that consists of 38 coding exons and encodes a polypeptide of 1500 amino acids. Based on function, *CPS1* could be divided into five sections, including the N‐terminal domain, bicarbonate phosphorylation domain (BPSD), unknown function subdomain (UFSD), carbamate phosphorylation domain (CPSD), and NAG‐binding domain (ASD). The N‐terminal domain could be further divided into intersubunit interaction domain (ISD) and glutaminase domain (GSD) corresponding to the small subunit of E. coli CPS.^13^ The mutations display a “private” nature between families, and specific relationship between genotype and phenotype is still under investigation, which further complicates the diagnosis.^3^

Currently, most reported cases were newborns in their first few days of life or adults, whereas cases that have their initial symptoms starting from late neonates to puberty are scarce.^11^, ^14^, ^15^ An approach to help establish diagnoses of CSP1D is needed from late neonate to puberty. In the present study, we report the clinical findings, management, biochemical data, molecular genetic analysis, and short‐term prognosis of five children with CPS1D.

## 2 MATERIALS AND METHODS

### 2.1 Subjects

We retrospectively studied CPS1D patients from the Children's Hospital of Chongqing Medical University from January 2014 to January 2019. In total, 130 patients were detected to have HA >100 μmol/L. Among them, 33 patients were suspected to have congenital metabolic diseases and genetic analysis was conducted. Eleven patients were diagnosed with OTC deficiency, and five of them were diagnosed with CPS1D. Their main clinical features and laboratory findings are summarized in Table 1. Blood taken for tandem mass spectrometry and urine gas chromatography tests were all in the acute phase. The study was conducted under the guidance of the Declaration of Helsinki 1975. All patients provided written informed consent for participation in the study, which was approved by the Children's Hospital of Chongqing Medical University Ethics Committee.

**Table 1:** Summary of clinical and biochemical features of CPS1D patients

Patient	Sex	Age of onset	Clinical presentation	Biochemical finding		EEG	MRI	Treatment		Current age and outcome
				Peak blood ammonia (μmol/L, nv 9‐33)	Plasma citrulline (μmol/L, nv 7‐35)			Acute phase	Maintenance phase	
P1	M	8 y 4 m	Confused status, lethargic, headache	261	6.79	N	N	Hemodialysis, arginine, citrulline, L‐carnitine, lactulose, protein restriction	Benzoate sodium arginine, citrulline, L‐carnitine, protein restriction	11 y Free of HA attacks, academic failure
P2	M	21 d	Vomiting, poor feeding, reduced consciousness, hyperventilation, limb hypertonicity	367.2	3.94	N	–	Arginine, citrulline, L‐carnitine, protein restriction	Arginine, citrulline, L‐carnitine, low‐protein diet, and powder milk	2 y 3 m Free of HA attacks, normal development
P3	M	11 y 9 m	Psychic hallucination, aggressive behavior, sleep disorder	218.4	5.55	A	N	Arginine, L‐carnitine, lactulose, protein restriction	Arginine, L‐carnitine, protein restriction	13 y Free of HA attacks, academic failure
P4	M	11 m 12 d	Reduced consciousness, suspected seizures	133.9	4.094	A	A	Arginine, L‐carnitine, lactulose, protein restriction	Arginine, L‐carnitine, protein restriction, liver transplant	4 y 8 m Free of HA attacks, moderate developmental delay
P5	F	2 y 3 m	Suspected seizures sleep disorder, Headache Aggressive, vomiting	222.2	4.644	A	N	Arginine, L‐carnitine, protein restriction	L‐carnitine, protein restriction	2 y 10 m Normal development

Abbreviations: A, abnormal; d, day; EEG, electroencephalogram; F, female; HA, hyperammonemia; M, male; m, month; MRI, magnetic resonance imaging; N, normal; y, year.

### 2.2 Mutational analysis

Genomic DNA was extracted from peripheral blood lymphocytes using QIAamp DNA Blood Mini Kit (Qiagen). Targeted next‐generation sequencing was performed according to experimental procedures previously described.^16^ All the genetic lesions detected were validated by Sanger sequencing. Sequencing results were aligned to the human genome (GRCh37/hg19) and compared with the established human *CPS1* sequences (National Center for Biotechnology Information Accession No. NM_001875). A group of 50 Chinese subjects without CPS1D were recruited as controls to test whether the mutations discovered were polymorphisms. Publicly available databases, such as the Single Nucleotide Polymorphism Database (dbSNP) (http://www.ncbi.nlm.nih.gov/SNP/), Human Gene Mutation Database (HGMD) (http://www.hgmd.cf.ac.uk/ac/index.php), and 1000 Genome Database (http://browser.1000genomes.org/index.html), were used to determine whether the mutations were known. The novel and known mutations were then assessed as disease‐causing using PolyPhen‐2 (http://genetics.bwh.harvard.edu/pph2/), Sorting Intolerant From Tolerant (SIFT, http://sift.bii.a-star.edu.sg/), and Mutpred2 (http://mutpred.mutdb.org/). Generation of protein plots was performed using Swiss‐pdb Viewer 4.10, and the Protein Data Bank entry codes used were 5DOU.

## 3 RESULTS

**Figure 1:**
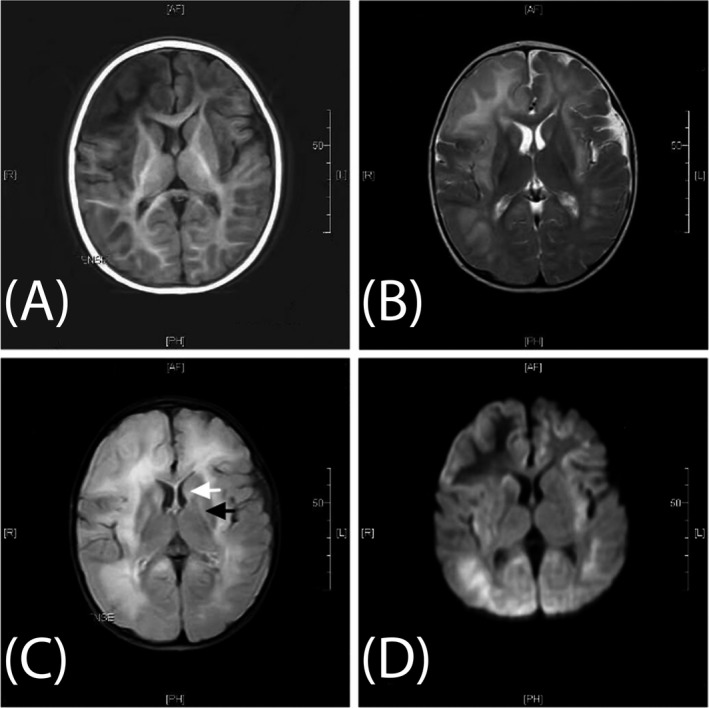
Brain magnetic resonance imaging of Patient 4 upon initial acceptance into the study. Brain MRI examinations were all performed at 1.5 T at the Children's Hospital of Chongqing Medical University. Axial T1 image (A), T2 image (B), fluid‐attenuated inversion recovery image (C), and diffusion‐weighted image (D) show diffused parenchymal edema. (A) Low signal intensity on T1‐weighted image (T1WI). (B) High signal intensity on T2‐weighted image (T2WI). (C) High signal intensity on FLAIR image involving the white matter, caudate head (white arrow), and bilateral putamina (black arrow). (D) High signal intensity on diffusion‐weighted image (DWI)

Table 1 summarizes the basic information and initial clinical presentation of 5 patients from non‐consanguineous parents. The patient's siblings and other family members were all healthy. The patient's peak blood ammonia level at presentation ranged from 133.9 to 367.2 μmol/L, and the liquid chromatography‐mass spectrometer/mass spectrometer (LC‐MS/MS) showed a decreased level of citrulline. Magnetic resonance imaging (MRI) and electroencephalogram (EEG) images of five pediatric patients are shown in Figure 1, Figures S1 and S2.

Patient 1 was referred to our Center for confusion, lethargy, and headache that occurred during febrile episodes beginning at 8 years old. The patient showed appropriate growth and normal psychomotor milestones, but with academic failure. Patient 1 experienced the same presentations 5 months before when suffering from suppurative tonsillitis, but the symptoms released after intravenous fluids were given and no specific tests were completed. The patient's blood ammonia decreased to normal range after positive management of hemodialysis, and L‐arginine infusion, L‐carnitine supplement, lactulose, and restrictive protein intake. Furthermore, long‐term therapy has been maintained with arginine, citrulline, L‐carnitine, and benzoate sodium. Currently, patient 1 is 11 years old, out of symptomatic onset with normal behavior, but still has an academic failure in primary school. Genetic analysis revealed the novel mutation c.478G > A (p.A160T) in exon 5 at the heterozygous level with the reported mutation c.1145C > T (p.P382L) in exon 11.^3^

Patient 2 became symptomatic at 21 days after birth, presenting with vomiting, poor feeding, reduced consciousness, rapid breath rate, reduced primitive reflexes, and hypertonic limbs. The patient was successfully treated with iv arginine, L‐carnitine, and adequate fluid supplementation. The re‐introduced protein with powder milk was carefully adjusted (started from 0.5 g/kg/day), and the patient tolerated up to 1.5 g/kg/day of protein when released from the hospital. Long‐term treatment included oral arginine, citrulline, and L‐carnitine, and ordinary powder milk was changed to a low‐protein diet as the patient has gotten older. No HA crisis has been observed. During patient 2's latest assessment at 2 years and 3 months old, he was able to communicate with simple words and motor development was normal. In patient 2, the frameshift mutation c.2865_c.2869delAAACT (p.T955Tfs*12) and the novel missense mutation c.3949C > T (p.R1317W) were found in *CPS1* gene.

Patient 3 showed reduced consciousness, psychotic behavior, and sleep disorder when he was 11 years old. A Level II hospital diagnosed the patient with autoimmune encephalitis and administered benzodiazepines to relieve aggressive behavior; however, the patient deteriorated to a coma and was transferred to our hospital. The patient had a history of learning difficulties and a tendency to avoid dietary protein. Electroencephalogram showed increasing 3‐5 Hz θδ background activity in the awaken state, while the MRI scan was normal. The patient's condition was stabilized after emergency management, including arginine, L‐carnitine, lactulose, and a low‐protein diet. At follow‐up, patient 3 proceeded to take oral arginine, L‐carnitine, and continue the dietary treatment; no metabolic crisis was reported. Currently, the patient is 13 years old with normal behavior, but shows academic failure and received retention in primary school. Molecular analysis of patient 3 revealed 2 heterozygous mutations in the *CPS1* gene, including c.1958T > G, which leads to the amino acid substitution p.V653G in exon 17, and c.1145C > T (p.P382L) in exon 1, which was also found in Patient 1.

Patient 4 exhibited reduced consciousness, poor feeding, and seizures during a febrile illness at 11 months of age. The MRI showed significantly diffused parenchymal edema and hyperintensities in fluid‐attenuated inversion recovery images involving the white matter, caudate head, and bilateral putamina. The EEG showed increased 2‐4 Hz δθ background activity in the awaken state mixed with atypical spikes in left occipital region. The boy was successfully treated with arginine, L‐carnitine, lactulose, and a low‐protein diet. At follow‐up, the patient's ammonia showed frequent elevation and he exhibited psychomotor retardation. The patient had a liver transplant at 4 years old. Thereafter, his blood anomia was relatively normalized with ordinary diet and no more medicines, but the psychomotor retardation is still obvious. During his latest assessment at 4 years and 8 months, he fails to walk steady and can only speak several simple words. In patient 4, the novel heterozygous nonsense mutation c.3945G > A (p.W1315X[186]) and the frequently reported missense mutation c.1760G > A (p.R587H) were detected in *CPS1* gene.^3^, ^9^, ^15^

Patient 5 was admitted to our center at 2 years and 4 months old. The patient presented with aggression, hard to sleep, vomiting, and suspected convulsion. The EEG connected during acute phase showed diffusion 3‐5 Hz δθ background activity during the awake state, combined with bursts of 3‐4 Hz δθ activity. The MRI was normal. The patient was successfully treated with arginine, L‐carnitine, and a low‐protein diet in acute phase and has continued to take oral L‐carnitine and keep a low‐protein diet in the subsequent clinical course. Currently, she is 2 years and 10 months old and psychomotor development is age‐appropriate. Two heterozygous missense mutations c.1271A > T (p.E379V) and c.3928C > T (p.P1265S) were identified in the *CPS1* gene.

Biallelic mutations in the *CPS1* gene were identified in all five patients and are summarized in Table 2. Sequencing data are shown in Figure S3. In total, nine mutations were detected, of which five were novel. None of the novel mutations we found have been listed in the public single nucleotide polymorphism databases (dbSNP) and have not been previously reported in HGMD and 1000 Genome Database. Of the total nine mutations, we identified seven missense mutations, one nonsense mutation, and one frameshift mutation. In silico prediction programs rate the changes as damaging.

**Table 2:** Mutations identified in CPS1D patients (mutations reported before are shown in bold type)

Patient	Exon	Nucleotide change	Amino acid substitution	CPS1 domain	In silico investigation			Highest MAF^d^	Population frequency^e^
					PolyPhen‐2^a^	SIFT^b^	MutPred2^c^		
P1	Exon5	c.478G > A	p.A160T	ISD	1	0.002	0.832	–	–
	Exon11	**c.1145C > T** f	**p.P382L**	GSD	0.865	0.015	0.518	<0.01	0
P2	Exon23	c.2865_c.2869delAAACT	p.T955Tfs*12	UFSD	–	–	–	–	–
	Exon33	c.3949C > T	p.R1317W	CPSD	0.998	0	0.882	–	–
P3	Exon1	**c.1145C > T** f	**p.P382L**	GSD	0.865	0.015	0.518	<0.01	0
	Exon17	c.1958T > G	p.V653G	BPSD	0.845	0.001	0.842	–	–
P4	Exon16	**c.1760G > A** g	**p.R587H**	BPSD	1	0	0.925	–	–
	Exon33	c.3945G > A	p.W1315X(186)	CPSD	–	–	–	–	–
P5	Exon11	**c.1271A > T** h	**p.E379V**	GSD	0.995	0	0.682	<0.01	1.163 e‐04
	Exon32	**c.3928C > T** h	**p.P1265S**	CPSD	1	0.001	0.735	<0.01	–

a PolyPhen‐2 grades the damaging effect of an amino acid substitution as “probably damaging” if the score is between 0.909 and 1, and “possibly damaging” if the score is between 0.447 and 0.908, and “benign” is the score is between 0 and 0.446.

b SIFT scores the substitution as ≤0.05 = damaging, which means that the change is predicted to affect protein function.

c MutPred2 scores the probability that the amino acid substitution is pathogenic. A score threshold of 0.50 would suggest pathogenicity.

d Highest minor allele frequency observed in any population including 1000 genomes phase 3, ESP, and gnomAD.

e The frequency of the East Asian population documented by the Exome Aggregation Consortium (ExAC).

f Ref.^3^

g Ref.^3^, ^9^, ^15^

h The mutation has been listed in the single nucleotide polymorphism databases (dbSNP) without a case report.

## 4 DISCUSSION

In the present study, we presented our main clinical findings, biochemical and molecular data from five segregated CPS1D probands and their parents. To date, more than 270 mutations have been reported and documented in HGMD (http://www.hgmd.cf.ac.uk/ac/index.php). However, to our knowledge as the country with the largest worldwide population, the cases reported in China remain scarce.^17^, ^18^ Most likely because of lack of awareness of the clinician and the access to testing, diagnostic, and life‐supporting facilities.^3^ With the first establishment of the guidelines for diagnosis and management of rare diseases on February 27, 2019, and the increasing use of gene technology, more CPS1D cases are accumulating.

Urea cycle disorders (UCDs) classically manifest themselves in the neonatal period and present with fatal HA. However, in recent years, non‐classical UCDs with atypical clinical courses have received more attention and are as frequent as those with classical presentations. Non‐classical UCDs mostly consist of f‐OTC and ASS deficiency, and usually present outside the newborn period, follow a mild and heterozygous course.^19^ Our research group has shared some clinical features of those cases, which has fulfilled the clinical spectrum of CPS1D. Physicians need to give more attention to atypical cases. In patients with recurrent gastrointestinal symptoms, change of consciousness, abnormal mental behavior, self‐selected low‐protein diet, unexplained developmental delay, or positive family history, blood ammonia should be a routine test.

Tempested correction of blood ammonia is critical for improving neurological prognosis.^5^ Treatment of CPS1D follows the recommendations for UCDs.^12^ Low‐protein diet should be administered with the replacement of arginine or citrulline, which could suppress the production of ammonia and promote the excretion of nitrogen.^20^ Calories given as carbohydrates and sufficient intravenous fluids should be ensured to maintain homeostasis while reducing catabolism of protein.^10^ Nitrogen‐scavenging agent, benzoate sodium, and phenylacetate sodium can conjugate glycine and glutamine to form atoxic compounds and be excreted from urine, which provide alternative pathways for excretion of excess nitrogen. Lactulose lowers the intestinal pH and causes intestinal hurry, thus to facilitate ammonia excretion.^20^ Probiotics create substrate shortage and inhibit the growth of other bacteria producing ammonia.^21^ Antibiotics, such as rifaximin, are used to inhibit intestinal bacteria to reduce ammonia production, but the high cost and accumulation effect are a limitation.^20^ Exogenous supplementation of L‐carnitine has been proposed to control HA, especially when evoked by valproic acid treatment.^22^ If HA persists despite all of these measures, dialysis should be instantly started.^5^ Early liver transplantation might shut down the patient episodes and result in better neurologic outcomes. For long‐term treatment, the plan needs to be individualized and should comprehensively consider the residual function of the enzyme, growth needs, environmental stress, and mental state. Our patients achieved fair ammonia control after the treatment combinations above were used. Although all patients were suggested to take sodium benzoate or sodium phenylbutyrate as the first‐line treatment for UCDs, only one patient is currently taking the drugs, probably because it is hard to obtain these drugs in China. Patient 4 is the only child in our group who underwent liver transplantation. Although the metabolic function was completely corrected afterward, developmental delay is still prominent since the transplantation was delayed by the scarcity of available livers.

Novel medicine and treatment approach are under research. Carglumic acid (N‐carbamoyl‐L‐glutamic acid, Carbaglu^®^, Orphan Europe) is an FDA‐approved orphan drug for the treatment of NAGS deficiency and is now under investigation for other inborn metabolic disorders. As an allosteric activator of CPS1, carglumic acid reactivates the genetically impaired UC and lowers systemic ammonia.^20^, ^22^, ^23^ In an induced hyperammonemic rat model, the flavonoid fisetin showed the ability to prevent oxidative stress, normalized blood ammonia, and increased the expression of UCD enzymes, including CPS1.^24^, ^25^ Hepatocyte or stem cell transplantation and new approaches for gene therapy have become promising treatment for CPS1D.^26^

Patient 3 was given phenobarbital to control his aggressive behavior when initially diagnosed with autoimmune encephalitis and his consciousness got worse and resulted in a coma. Phenobarbital could aggravate UCDs. Other drugs reported to associate with hyperammonemic decompensation are valproate and L‐asparaginase/pegaspargase, topiramate, carbamazepine, phenobarbitone, phenytoin, primidone, furosemide, hydrochlorothiazide, and salicylates.^1^ In addition, full intravenous amino acids can worsen the condition and should not be given for potential UCD patients with gastrointestinal symptoms or issues with consciousness.^17^

We could not make a prognosis because of the short follow‐up. Waisbern et al reported that CPS1D was associated with global early developmental delays and autism spectrum disorder, but the sample size of the study was small.^27^ One of our patients (Patient 4) showed moderate psychomotor retardation in coordination with severe neuroimaging changes during acute phase, and another two patients (Patient 1, Patient 3) had difficulty studying, but no behavior problem was reported by their parents.

Similar to previous reports,^3^ most of the mutations in our study were compound heterozygous and mainly consisted of missense mutations. The pathogenic role of mutations leading to aberrant proteins is widely understood. In our group, Patient 2 carried a frameshift mutation and Patient 4 carried a nonsense mutation; both had relatively early onset, which may be due to the strong pathogenicity of the enzyme truncation mutations. However, the disease‐causing roles in most missense mutations have not been proven.^3^, ^6^, ^28^ The pathological effect might be explained when the mutations affect residues predicted to interfere with the catalytic sites, the internal tunnel, or the regulatory domain.^6^

The mutations c.478G > A (p.A160T) and c.1145C > T (p.P382L) were observed in the N‐terminal domain. The function of this domain is unclear in human *CPS1*, but it is hypothesized to activate the catalytic activity and stabilize the enzyme.^28^ Pekkala et al found that the activities of the p.S123F and p.H337R mutant forms were reduced by approximately 60% and 70%, respectively, in recombinant baculovirus–insect cell rat liver CPS1,^29^ indicating that these mutations may not completely inactivate CPS1 enzyme. These results may explain the attenuated clinical manifestations and the high sensitivity of diet therapy in Patient 1.

**Figure 2:**
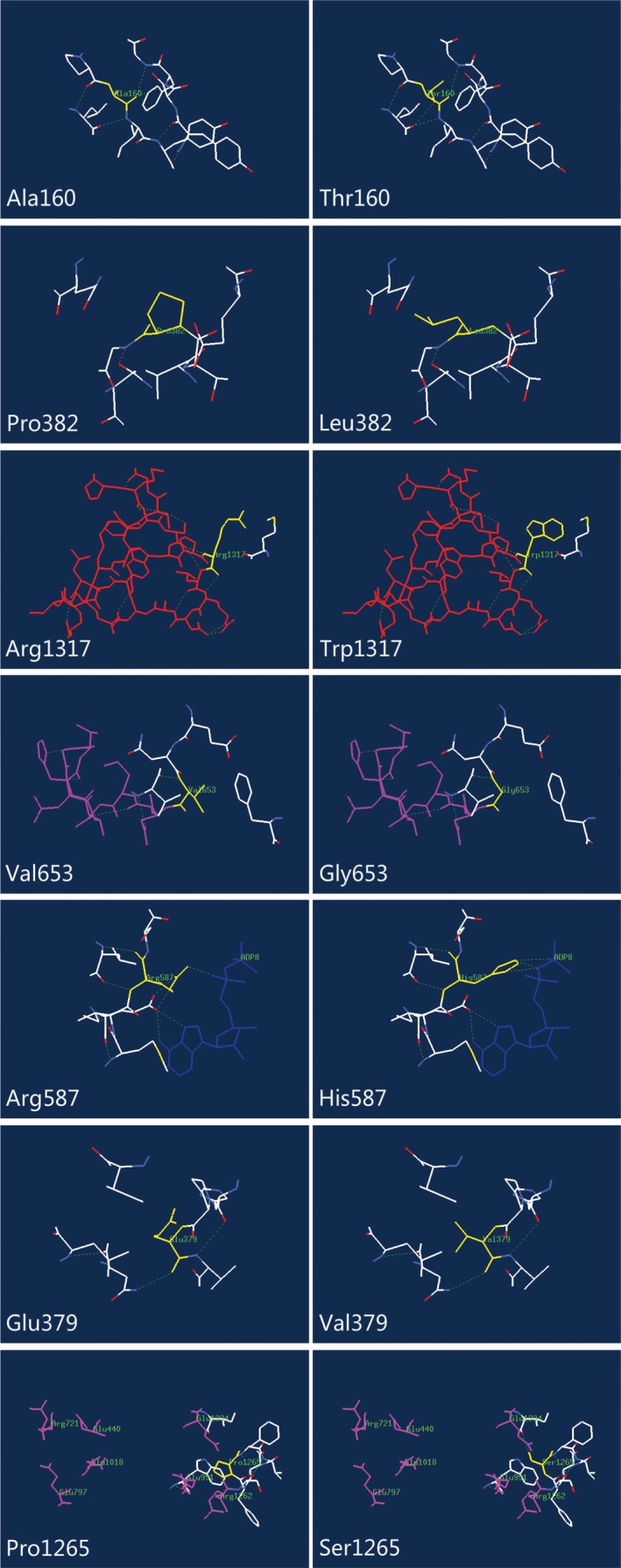
Structural analysis of wild‐type (WT) and the variant *CPS1* with mutations. The residues of missense mutant sites together with the nearby functional site are illustrated in WT and variant *CPS1* using Swiss‐Pdb Viewer. The computed hydrogen bonds are shown as green dashed lines. Residues of the mutant sites are highlighted in yellow. T'‐loop (L3β15‐L3β16 loop, residues 1311‐1333) is shown in red, K‐loop (L1β11‐L1β12, residues 654‐662) is shown in purple, molecular ADP is shown in deep blue, and seven amino acids contributional to the postulated carbamate tunnel are highlighted in pink. The nearby residues within 5 Å are shown in white

The genetic lesion c.3949C > T (p.R1317W) was identified in Patient 2 at a heterozygous level with frameshift mutations c.2865_c.2869delAAACT (p.T955Tfs*12). p.T955Tfs*12 introduces a stop codon in an UFSD, which connects the BPSD and CPSD. The UFSD has been proposed to be an important region for proper enzyme folding and for regulatory cross‐talk between NAG and phosphorylation sites.^13^ p.R1317W belongs to T'‐loop (L3β15‐L3β16 loop, residues 1311‐1333), which is a hub for NAG signal transmission to the catalytic machinery.^30^ As predicted by Mutpred2, introduction of a hydrophobic residue at a positive charge position may alter ordered interface and changed DNA binding. Figure 2 shows the structure of the T'‐loop; the mutations affect the protein structure by changing the hydrogen bonding and the spatial conformation. Therefore, the c.3949C > T (p.R1317W) and c.2865_c.2869delAAACT (p.T955Tfs*12) mutations may explain the neonatal onset and severe clinical manifestations observed in our patients.

p.V653G is located in the BPSD close to K‐loop (L1β11‐L1β12, residues 654‐662). K‐loop has a coordinated potassium ion in its center and is involved in intimate interactions with bound ADP.^30^ The mutation is predicted to alter the metal binding, causing it to fail to catalyze the bicarbonate‐dependent ATPase partial reaction. Later onset of this patient suggests that either the patient has a functional copy in the other allele or that the mutations have a less severe effect.^7^

The p.R587H is located within CpG dinucleotides, which is thought to have a mutation rate 13‐fold higher than the remaining coding sequence. p.R587H affects the residue of the catalytic site and has been shown to practically abolished enzyme activity by blocking carbamate synthesis.^31^ Another mutation found in Patient 4 was a nonsense c.3945G > A (p.W1315X[186]) that caused a truncated protein resulting in a lost ASD. N‐acetylglutamate triggers the long‐range conformational changes in CPS1 and, in its absence, CPS1 exhibits ≤2% of the activity at NAG saturation.^30^ The loss of NAG affinity is pathogenic; therefore, this finding in patient 4 could explain his recurrent episodes and worst neuropsychological outcome. In vitro glycerol has been proven to activate CPS1 without binding to the NAG‐binding site,^28^ indicating that the patients carrying this mutation could possibly be treated with designed pharmacological chaperones in the future.

The genetic lesion c.1271A > T (p.E379V) in Patient 5 located in the N‐terminal domain. The substitution is predicted to alter the metal binding and interfere with the catalytic property of CSP1. In the second allele, we identified c.3928C > T (p.P1265S) at the heterozygous level. The lesion was located at the CPSD and adjacent to the possible carbamate tunnel. Carbamate migrates between the phosphorylation active centers through a water shielded tunnel to the active the center of the CPSD, where it is phosphorylated to carbamoyl phosphate.^30^ Substitution of polar residues from non‐polar residues may hamper the tunneling of intermediates. Neither mutations were interpreted to abolish the enzyme activity, which mirrored with the mild clinical observations from the patient 5. Long‐term follow‐up is still needed to evaluate the patient's biochemistry and neuropsychological changes.

## 5 CONCLUSION

In the present report, we present five children diagnosed with CPS1D. Genetic analysis revealed five new mutations that have not been previously reported, thereby expanding the mutational spectrum of the *CPS1* gene. The overall clinical manifestations and biochemical data of our patient group were consistent with the variation of gene mutations, indicating that the current understanding of the CPS1 protein structure could be used to interpret unverified missense mutations. Our report aims to improve physician's awareness of CPS1D to aid early diagnosis and intervention, thus improving neurological outcomes in these patients.
