# Supplementary figures and images for: Molecular, biochemical, and clinical analyses of five patients with carbamoyl phosphate synthetase 1 deficiency
Source: J Clin Lab Anal. 2019 Nov 20;34(4):e23124. doi: 10.1002/jcla.23124 (PMC7171324; doi:10.1002/jcla.23124)

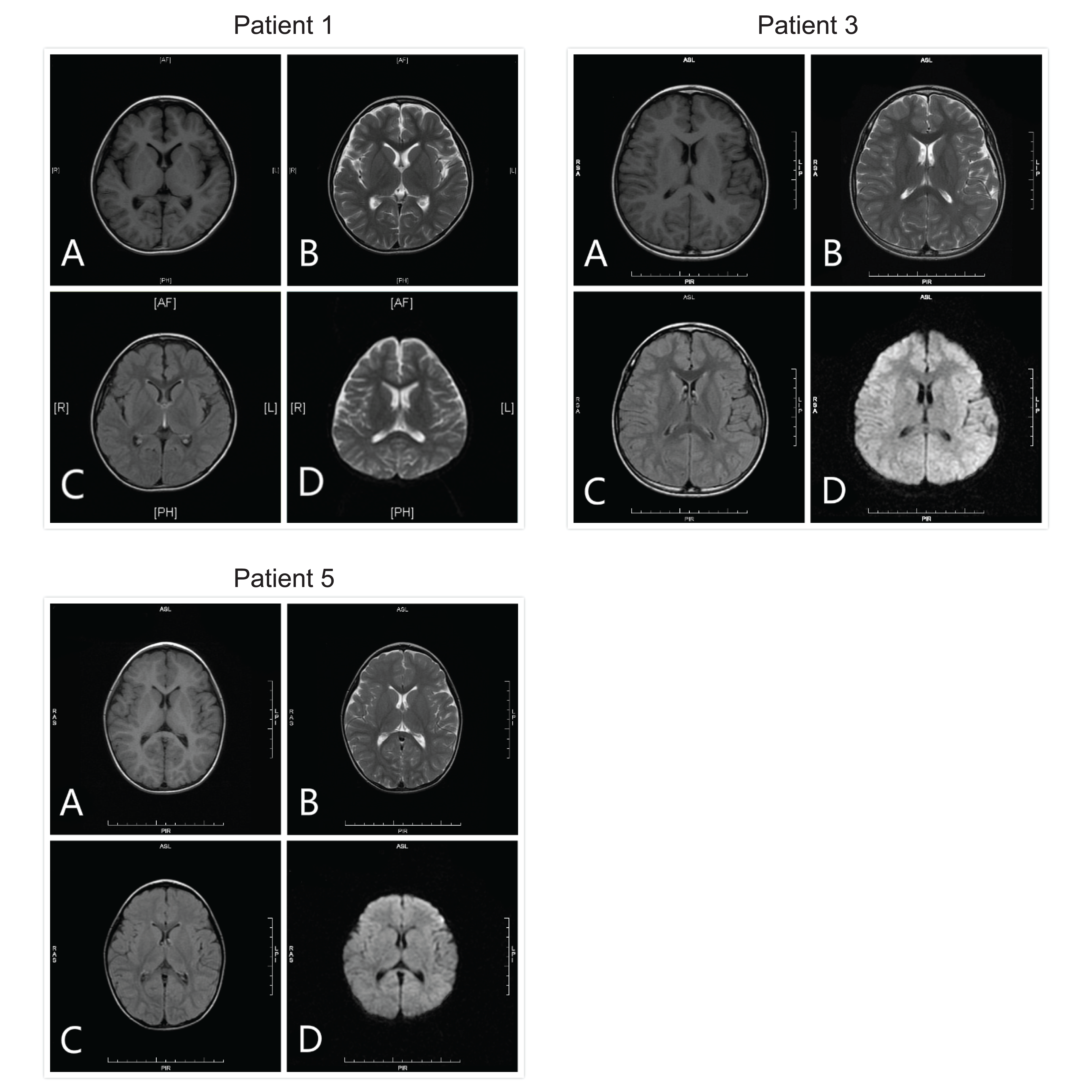

Supplement: Supplementary file 1 [file JCLA-34-e23124-s001.tif]

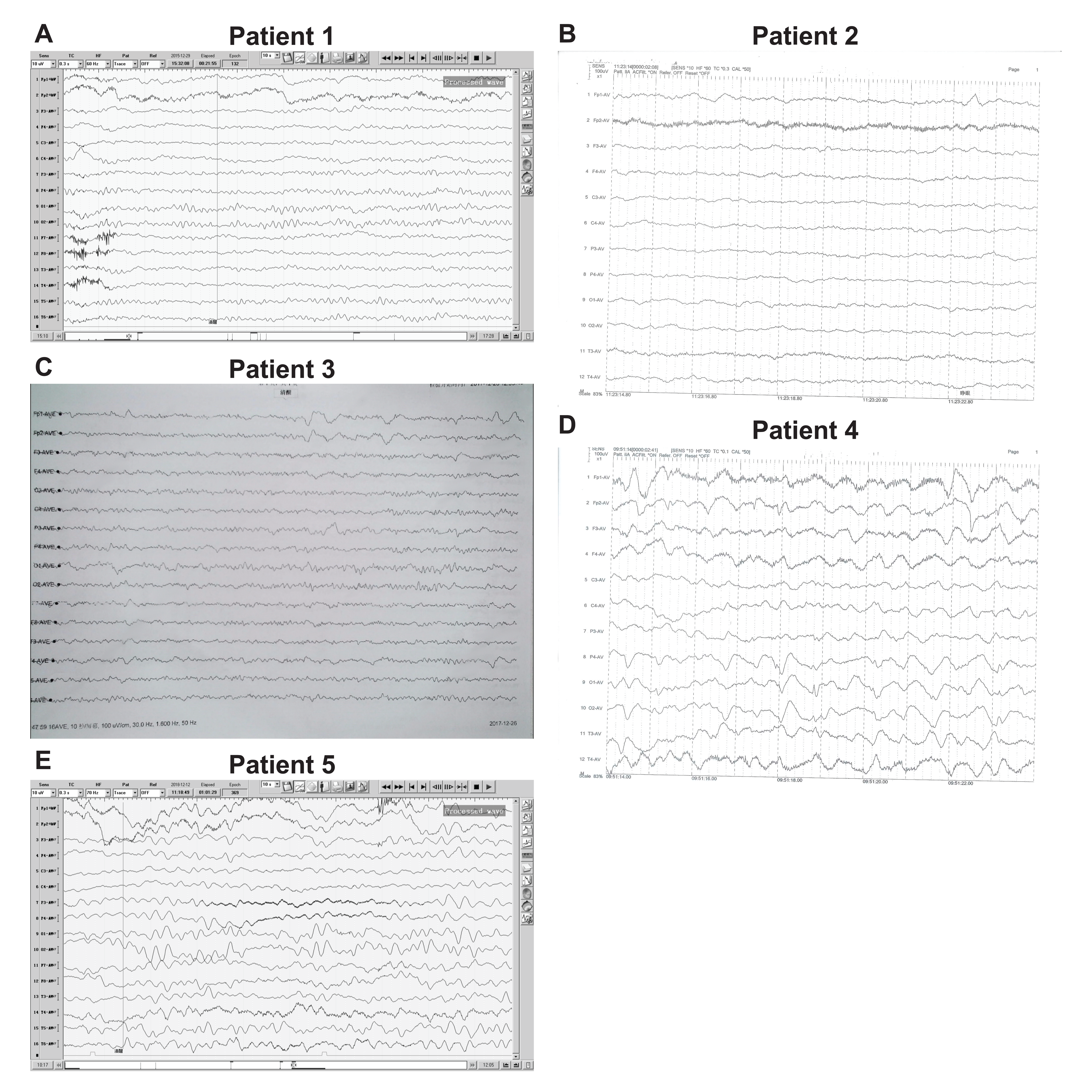

Supplement: Supplementary file 2 [file JCLA-34-e23124-s002.tif]

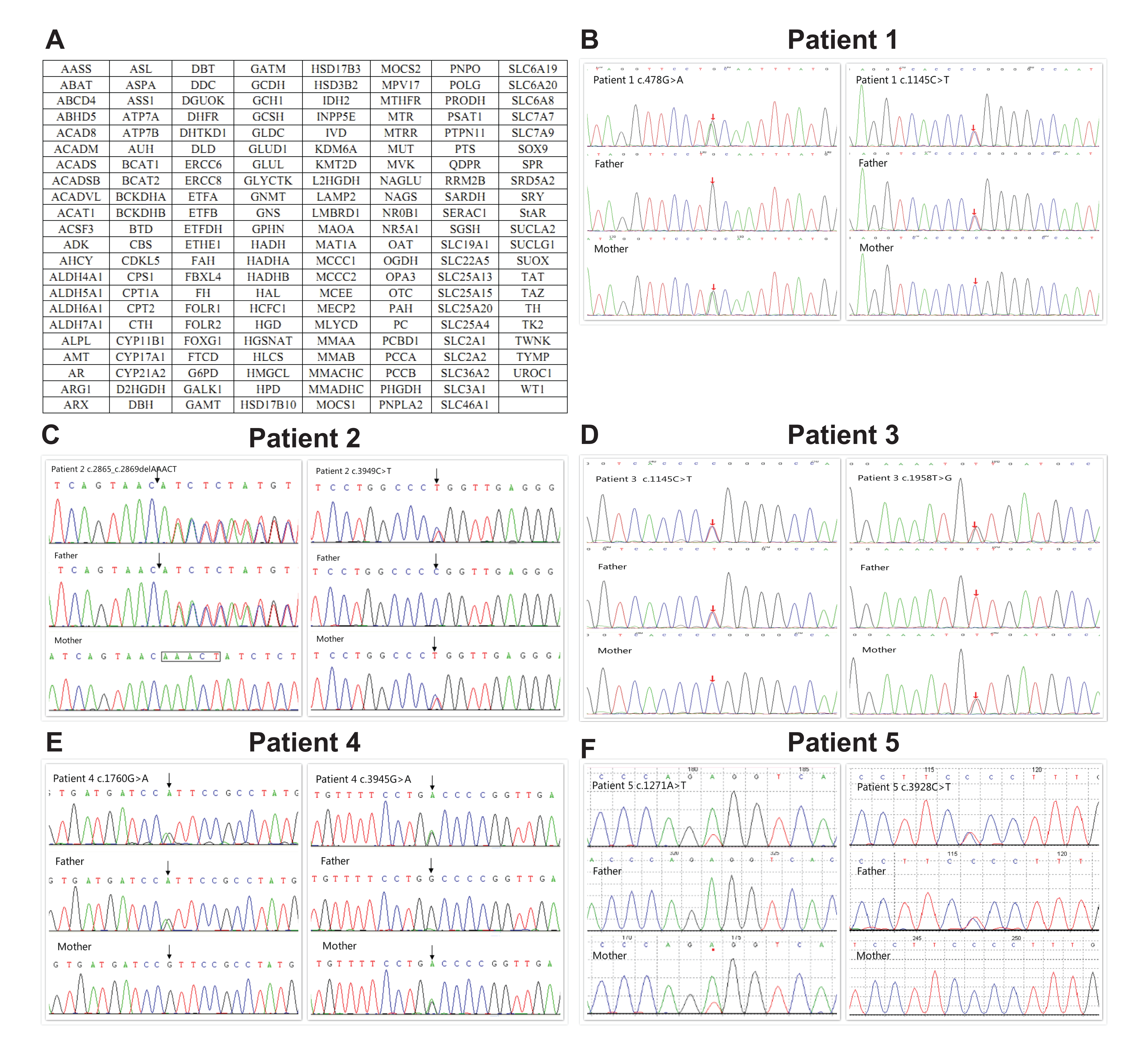

Supplement: Supplementary file 3 [file JCLA-34-e23124-s003.tif]
